# Localization and segmentation of atomic columns in supported nanoparticles for fast scanning transmission electron microscopy

**DOI:** 10.1038/s41524-024-01360-0

**Published:** 2024-08-03

**Authors:** Henrik Eliasson, Rolf Erni

**Affiliations:** 1 Electron Microscopy Center, Empa – Swiss Federal Laboratories for Materials Science and Technology, Überlandstrasse 129, 8600 Dübendorf, Switzerland; 2 Department of Materials, ETH Zürich, CH-8093 Zürich, Switzerland

**Keywords:** Nanoparticles, Characterization and analytical techniques, Heterogeneous catalysis, Transmission electron microscopy

## Abstract

To accurately capture the dynamic behavior of small nanoparticles in scanning transmission electron microscopy, high-quality data and advanced data processing is needed. The fast scan rate required to observe structural dynamics inherently leads to very noisy data where machine learning tools are essential for unbiased analysis. In this study, we develop a workflow based on two U-Net architectures to automatically localize and classify atomic columns at particle-support interfaces. The model is trained on non-physical image simulations, achieves sub-pixel localization precision, high classification accuracy, and generalizes well to experimental data. We test our model on both in situ and ex situ experimental time series recorded at 5 frames per second of small Pt nanoparticles supported on CeO_2_(111). The processed movies show sub-second dynamics of the nanoparticles and reveal site-specific movement patterns of individual atomic columns.

## Introduction

The shape and structure of supported nanoparticles are of great importance in applications like catalysis where the nanostructure defines the available active sites. Small nanoparticles are not static, and given energy, they can undergo a wide range of reconfigurations, changing their properties as a consequence^1–3^. Time-resolved high-resolution scanning transmission electron microscopy (HR-STEM) data showing dynamics of such species could potentially reveal properties of specific structures and ways of promoting their formation. However, such data are rare and inherently noisy due to the low electron dose required to ensure the nanoparticle’s structural integrity during imaging, calling for advanced post-processing to extract information from the data.

The first problem in many HR-STEM processing pipelines is to accurately localize the imaged atomic columns. Sub-pixel accuracy is crucial as the column positions are the foundation of most subsequent analyzes, whether it be strain mapping, calculation of physical properties, atom counting, or other^4–8^. Standard semi-automatic peak finding techniques do not cope well with the increased Poisson noise that high frame rates and/or low electron dose bring, typically leading to poor localization precision and false predictions. Furthermore, assigning element labels to atomic columns in noisy data can also be non-trivial as the Z-contrast of the high-angle annular dark-field (HAADF) signal is degraded by noise. Thus, to study sub-second dynamics of atomic columns, more sophisticated analysis methods must be consulted.

Deep convolutional networks are extremely powerful for various problems within computer vision and image processing^9–12^. The U-Net^13^ in particular, is an architecture that has found many applications within the TEM community. Whether it be segmentation of nanoparticles, atomic column localization, denoising and super resolution, or more specific problems, the U-Net finds a natural place in most processing pipelines^14–24^. One of the earliest applications of a U-Net to study electron microscopy images of nanoparticles was presented by Madsen et. al. in 2018^19^. Since then, several libraries have become openly available offering high-accuracy column localization in STEM images, two of the more prominent being AtomSegNet^18^ and AtomAI^25^.

The *Segmentor* model in AtomAI allows for both localization and segmentation of atomic columns. However, this model has thus far mostly been applied to images of 2D materials or highly periodic lattices covering most of the image, and is unproven on images of particle-support interfaces where different lattice geometries meet and a large portion of the image may contain background^26–33^. Furthermore, using a single U-Net to tackle both the task of peak finding and segmentation, as the *Segmentor* does, imposes limitations on the type of ground truth that can be used to encode the sub-pixel positions, and can complicate model training for difficult tasks. It also makes the segmentation dependent on the pixel values of the input images which puts a high demand on the training data to mimic the experimental data, limiting its broader applicability.

**Fig. 1:**
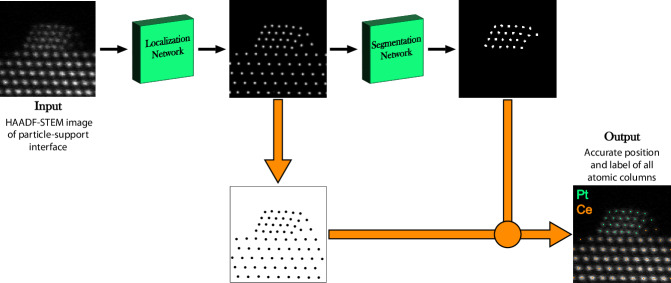
Model overview. An overview of the model used in this work to automatically detect and classify atomic columns in HAADF-STEM images of particle-support interfaces. The model takes a grayscale image as input and outputs a point set of predicted column positions with a corresponding label, particle (1) or support (0). The localization network is trained on simulated data with superpositions of gaussians as ground truth. The predicted positions are extracted by calculating the center of mass of each separated bright region in the output map following a threshold operation. The output image is then binarized and used as input for the segmentation network which has been trained to remove the regions corresponding to the support columns.

In this work we present a neural network based on two individually trained U-Net architectures for the fully automatic localization and classification of atomic columns in HAADF-STEM images of particle-support interfaces (Fig. 1). The model is trained on non-physical synthetic data and we evaluate its localization precision, robustness to noise and classification accuracy. To investigate our model’s generalizability, we also apply it to experimental time series recorded at 5 frames per second (fps) of the common oxidation catalyst Pt/CeO_2_, both ex situ and in situ using a gas-cell holder.

## Results and discussion

### Generating synthetic data

A large and varied dataset of simulated images with known sub-pixel coordinates of all atomic columns is necessary to quantitatively evaluate any column-finding technique. Physically accurate image simulations are computationally expensive, and the benefits of a large dataset for model training makes simpler, non-physics-based approaches to image simulation appealing. Our approach begins with the generation of a set of column positions distributed in a pattern resembling a small nanoparticle on a support where the points of the particle region and the support region follow two distinct lattice geometries. This is followed by image formation where columns are modeled as bivariate Gaussians, and a handcrafted algorithm for noise application finalizes a semi-realistic-looking 128 × 128 image in less than a second. Many parameters like lattice geometry, lattice spacing, background intensity, noise level, column sizes, and column intensity are randomized to ensure a wide variety in the training data.

**Fig. 2:**
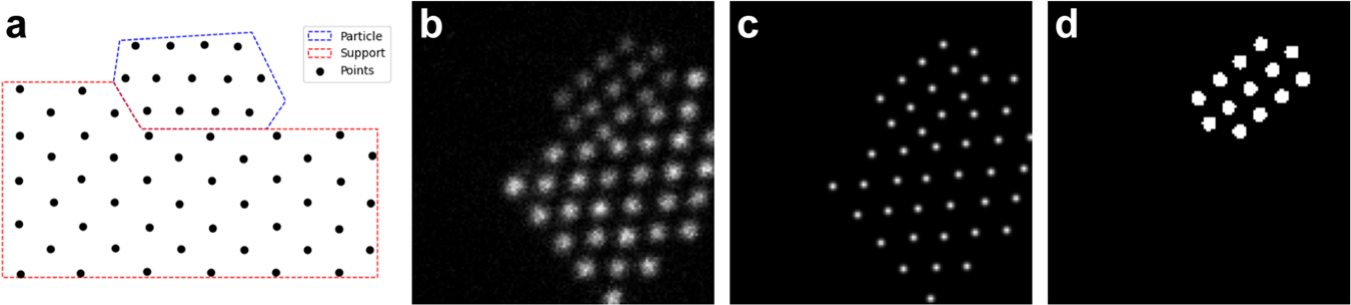
Synthetic data. **a** The point set is created by combining the subsets of particle and support, each generated in their respective polygon. The points within each region follow a specific lattice geometry with small displacements applied to each column. **b** The generated image. **c** The localization ground truth: a superposition of normal isotropic Gaussian profile placed at each sub-pixel position. **d** The binary mask used to generate the ground truth for the classification network from the output of the localization network. Each pixel with center within 3 pixels Euclidian distance of a column position is set to 1.

In order to generate the ground truth point set, the outline of the particle and support regions are first defined. The interface is shared by both regions and is defined by 3 interface-points in a wedge shape with a random angle between 90° and 180°, drawn from a beta distribution (α = 1, β = 3, skewed towards a flat interface), for a chance to generate step edge interfaces. The support polygon is formed by these 3 interfacial points plus 4 additional points that close the polygon in a rectangular manner (Fig. 2a). The particle polygon is formed by the interface points plus 3-5 randomly sampled points from the perimeter of a semi-circle drawn between the outer interface-points. Points following a hexagonal, oblique, or square Bravais lattice with a random lattice constant and rotation are generated within the regions. The ground truth point set is the union of these two subsets.

Each point is modeled as a bivariate Gaussian and the initial image is formed as the superposition of such Gaussians placed at each point in the point set. The covariance matrix of each Gaussian is set as

$$\varSigma =\left(\begin{array}{cc}a{\lambda } & b\\ c & a{\lambda }\end{array}\right),$$

where *a* is the lattice constant, *λ* is a scale factor, and *b* and *c* are random numbers generated from a uniform distribution over the interval [-1,1]. The same covariance matrix is used for all columns within the same image. Column intensities are set differently for particle columns and support columns. For particle columns, the intensity is set as

$${I=I}_{\min }+\left(1-{I}_{\min }\right)\frac{1}{1+{e}^{k\frac{d}{{d}_{\max }}}},$$

where I_min_ is the lowest possible intensity, *d* is the distance from the column to the middle of the particle interface, *d*_max_ is the largest distance of any particle column to the interface, and *k* is the decay rate which is uniformly sampled between 0 and a specified maximum value. For the support columns, the intensity is either set by a random number sampled from a uniform distribution, or by distance to the edge of the support. These schemes are used to mimic decaying intensity as the substrate gets thinner. The noise profile is a combination of Gaussian, Perlin, and Poisson noise, and is applied to the superposition of Gaussians in a handcrafted order. Perlin noise is used to generate medium-to-large regions of varying intensity, mimicking out of focus objects or contamination in HAADF-STEM images. The perceptual noise level of our simulated images is determined by a factor which scales the pixel values of the image array before Poisson noise is applied. Scan distortions were also applied to some images by randomly shifting rows by a given distance. A subset of the simulated images is displayed in Supplementary Fig. 1.

Two complementary ground truth images are also simulated along with every image (Fig. 2c, d). For the localization task, a superposition of isotropic Gaussians placed at each ground truth sub-pixel position, and for the segmentation task, a binary mask where all pixels with center within 3 pixels Euclidian distance of a column position is set to 1. This mask is later used to mask out the particle columns from the output of the localization network forming the ground truth for the segmentation network training.

In total, a dataset of 10000 images was generated as training data for the localization and classification networks. To benchmark the performance of the model as a function of noise, a separate dataset of 500 unique images, each in 12 variations with increasing noise level, was generated. The noise levels were selected by scaling the pixel values of the image before Poisson noise application such that the structural similarity (SSIM)^34^ compared to the highest signal image decreased with steps of about 0.05 from 1 to 0.45.

### Detecting atomic columns

**Fig. 3:**
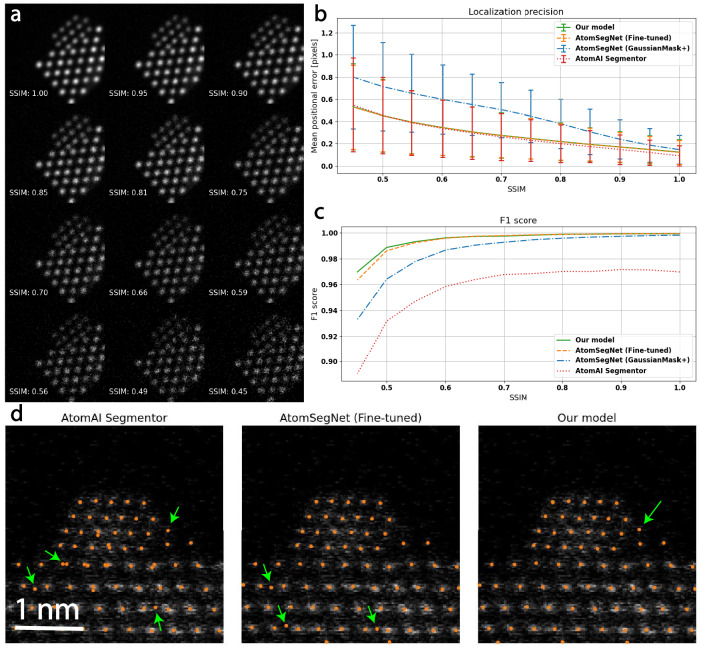
Evaluation of the localization network. **a** An example image from the test set. The models are evaluated over 500 unique images where each image comes in 12 distinct noise levels with structural similarity (SSIM) dropping in steps of about 0.05 from 1 to 0.45. **b** Localization precision as a function of SSIM level. AtomSegNet performs significantly better after fine-tuning on our dataset, achieving comparable precision to our model. Sub-pixel precision can be expected at each investigated noise level, with a precision of 0.12 pixels for high signal data and 0.53 for the highest noise level. AtomAI’s *Segmentor* model achieves the lowest positional error out of all models for high signal data (0.09 pixels). The error bars indicate one standard deviation. **c** The F1 score of the models as a function of SSIM level. Our model slightly outperform the fine-tuned AtomSegNet at higher noise levels (0.970 against 0.963). The F1 score of all models drop significantly at the highest noise levels, indicating an increasing number of false predictions. The output of the *Segmentor* model is inferior, mainly due to double predictions in the same column and false negatives close to the image border. **d** The models applied to a low-dose *128×128* experimental image of a Pt nanoparticle supported on CeO_2_. The fine-tuned AtomSegNet and our model exhibit comparable performance, where the only difference is a few disputable false predictions. The *Segmentor* model struggles with double predictions and some disputable false positives.

A U-Net architecture was employed to predict the sub-pixel position of atomic columns in HAADF-STEM images (Fig. 3a). The U-Net is 4 levels deep with concatenation skip connections between the encoder side and the decoder side. The model takes a single-channel image as input and increases the number of feature maps at each level as: 64, 128, 256, 512 (Supplementary Fig. 2). Each convolutional layer uses a kernel size of 3 × 3. Down-sampling is done by max-pooling, reducing the spatial dimension by a factor of 2 at each level. A sigmoid output layer is used to output a grayscale prediction in the range [0,1]. Simulated data were randomly split into training and validation set with 7500 and 2500 images, respectively. The data were not augmented in any way except for min-max normalization. The model was trained using the Adam optimizer, a dynamically weighted MSE (mean squared error) loss function, a batch size of 128, and a cosine annealing scheduler starting at a learning rate of 0.0005 decaying down to 0.0001. We dynamically weight the contribution of false positives and false negatives to the loss function to overcome a dataset imbalance in the early epochs and to suppress false positives at the later stages of training. As a significant portion of the pixel values in most training images are close to 0 (vacuum background), false negatives are punished in the first 4 epochs of training to avoid getting stuck in the local minima of outputting all-black prediction maps. This is done by scaling the contribution to the mean square error in the ground truth regions that have a pixel value larger than 0.1. In the same way, we punish false positives for the remaining epochs to limit false predictions. The model output is thresholded at 0.1 and each separated bright region in the predicted map is treated independently to decode its corresponding sub-pixel position. Using a superposition of Gaussians as ground truth, the sub-pixel position of each region is extracted by center of mass (CoM).

To evaluate the robustness of our localization model and benchmark it against existing solutions like AtomSegNet and AtomAI, the test dataset of 500 unique images, each in 12 noise variations, was used. We opted to benchmark our model against AtomSegNet with the *GaussianMask+* model weights as they have been found optimal in previous work^35^, and the *Segmentor* model in AtomAI with *nb_classes* = *3* as it was designed to solve a similar problem to ours. We also fine-tune AtomSegNet by training it on our training data for 100 epochs with a cosine annealing learning rate scheduler (*lr* = 0.001− > 0.00001) and a standard MSE loss. The *Segmentor* model was trained for 3000 epochs with a linearly decaying learning rate from 0.001 to 0.00001 and stochastic weight averaging enabled.

We define a false positive as a predicted position not being the closest predicted position within 3 pixels of any ground truth position, and a false negative as no predicted position being within 3 pixels of a ground truth position. As the GaussianMask+ AtomSegNet and *Segmentor* was trained on 256 × 256 images, we resize the test data from 128 × 128 before passing it through those models. The Cartesian coordinates of the predicted positions are then divided by 2 for the pixel error to be comparable with the models trained on 128 × 128 data. Localization precision and F1 score of the models are presented in Fig. 3.

In terms of localization precision, all investigated models except for the GaussianMask+ AtomSegNet perform comparably well, with the *Segmentor* model performing slightly better on high-signal data and slightly worse on very noisy data. Looking at the F1 score, however, we note that the *Segmentor* model suffers from a substantial number of false predictions, which upon manual inspection originates mainly from double predictions within the same column and missed columns at the image border. We note that our model achieves a slightly higher F1 score than the fine-tuned AtomSegNet for the highest noise levels investigated. The fine-tuned AtomSegNet model performs significantly better than AtomSegNet with the *GaussianMask+* weights. By manual inspection, we find that the F1 score of the *GaussianMask+* model suffers from false negatives in the particle regions, and we believe that the worse localization precision is due to a bias arising from the test set being simulated in a similar way to the training data used to the fine-tuned model.

The models were applied to experimental time-resolved HR-STEM data of small Pt nanoparticles supported on CeO_2_ to evaluate their generalization. The qualitative difference here between the fine-tuned AtomSegNet and our model is minor in standard Poisson-dominated noise environments, manifesting itself only in isolated, disputable predictions. The *Segmentor* model suffers from the same problems as it did on the training data (Fig. 3d). To explore another type of noise, we apply the models to similar data as before but acquired in situ with the DENSsolutions Climate holder (see methods).

**Fig. 4:**
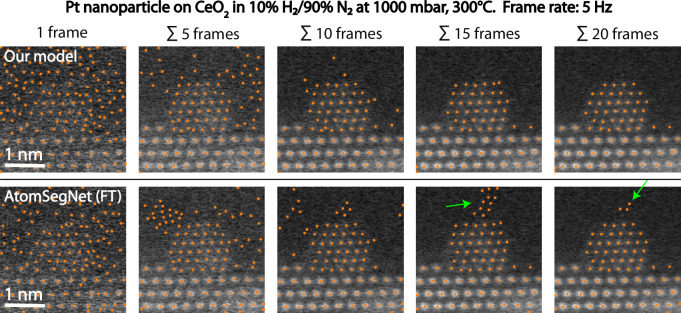
Column-detection in gas-cell data. Our model and the fine-tuned AtomSegNet model were evaluated on experimental data recorded in situ with the DENSsolutions Climate holder. The raw data are a time series recorded at 5 frames per second of a Pt nanoparticle supported on CeO_2_(111) in 10% H_2_/90% N_2_ at 1000 mbar, 300 °C. Our model stands out as the more robust one, and only 15-20 frames have to be summed to avoid false predictions in the background.

Due to the poor generalization of the *Segmentor* model, we now focus only on our model and the fine-tuned AtomSegNet. We evaluate the models’ robustness to the “in situ noise” by applying them to the raw data and 4 time series generated from the raw data where each frame is the sum of itself and the subsequent n frames, with n = 5, 10, 15, 20. This not only gives us a way of evaluating the robustness to noise, but also an idea of how much the temporal resolution is compromised in in situ experiments. The outputs of our model and the fine-tuned AtomSegNet from the first image in each series is displayed in Fig. 4. Here our model stands out as more robust, needing only 15–20 frames to be summed to avoid false predictions in the background, while AtomSegNet still struggles after 20. If false predictions in the background region are of little concern, most particle columns seem to be identified already at 5–10 summed frames. This indicates that about a 10–20 times higher beam dwell time is needed to extract the same type of information in situ as we could ex situ. It should also be noted that a higher beam current of 64 pA was used for this particular in situ experiment compared to the 20 pA used for the ex situ data in this work (see Methods for all imaging parameters), making a factor of 10–20 a lower estimate.

In conclusion, while all models achieve a comparably low localization error over the whole noise range, ours stand out as the most robust when evaluated on simulated data, ex situ data, and in situ data. Furthermore, our localization network also generalizes well to larger input images and images from other microscopes than our own (Supplementary Fig. 3).

### Labeling atomic columns

In noisy HAADF-STEM images, labeling atomic columns into either particle or support can be non-trivial, particularly for particles at step edges. A U-Net architecture was also employed for this task. The U-Net is 5 levels deep with concatenation skip connections between the encoder side and the decoder side. The model takes a binarized version of the output from the localization U-Net as input and increases the number of feature maps at each level as: 128, 256, 512, 1024, 2048 (Supplementary Fig. 4). Down-sampling is done by average-pooling, reducing the spatial dimension by a factor of 2 each level. The convolutional block includes batch normalization after each convolutional layer and a dropout layer (50%) at the end. All convolutional layers use a 3 × 3 kernel. A sigmoid output layer followed by binarization with a threshold at 0.5 is used to output a binary mask where only the particle regions remain bright. The ground truths for training are the intersection between the ground truth masks from the image simulation (Fig. 1d) and the binarized version of the outputs from the localization U-Net. The data were randomly split into training and validation set with 7500 and 2500 images, respectively. The model is trained using the Adam optimizer, a standard binary cross entropy loss function, a batch size of 32, and a constant learning rate of 0.000005.

We benchmark our model against the *Segmentor* model. Model performance was quantitatively evaluated on the test set by calculating the F1 score of the labels assigned to correctly identified columns from the localization task. A small second test set of 9 manually annotated experimental images was also used to qualitatively evaluate the models (Supplementary Fig. 5 and Supplementary Fig. 6).

For our model, all predicted column positions are assigned a label by sampling the pixel value of their coordinate in the binarized segmentation output. Our model performs well on the simulated test set, achieving a high F1 score at all noise levels, decreasing slightly at higher noise levels from about 0.968 to 0.947 (Fig. 4) due to degradation of the localization model’s output. Most false predictions are found at the interface between particle and support, where arguably the most difficult columns to label are positioned. We therefore also calculate the F1 score of the interface columns alone. We define interface columns as those positions in the Delaunay triangulation of the entire set of predicted positions that have a neighbor of the other class. The F1 score of the interface columns follow the general appearance of the full F1-score but shifted down by about 0.005 at low noise levels and 0.01 at the highest noise level investigated. On the manually annotated test set, our model achieves a classification accuracy of 99.43%. Our segmentation model performs well on many experimentally observed particle-support interfaces including oblique-on-oblique (Fig. 4c), oblique-on-square (Fig. 4a), but also irregular interfaces as seen in Fig. 4b. However, the model does not generalize well to larger input images (Supplementary Fig. 7).

**Fig. 5:**
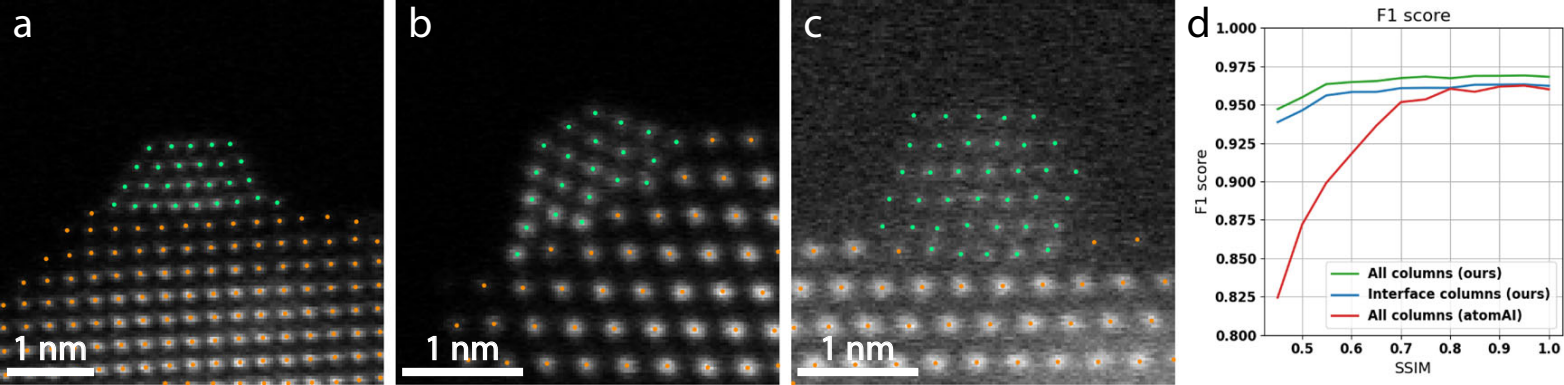
Evaluation of the classification model. Output of the segmentation model on an (**a**) oblique-on-square interface, (**b**) irregular interface, (**c**) oblique-on-oblique interface imaged in situ. **d** F1 score of the segmentation network, the interface columns are the most difficult for the model to predict correctly.

The *Segmentor* model proves far less robust to noise compared to our model, and its F1 score plummets from an initially acceptable value of 0.961 for high-signal data to 0.825 for highly noisy data. We can only speculate about the reasons for this but it is likely due to trying to solve both the localization task and the segmentation task with a single model. This makes the segmentation task directly dependent on the pixel values of the noisy input image. With our approach this is avoided, as the input to the segmentation model is always binary. However, a benefit of the *Segmentor* model is that it can segment the image into more than two classes, something our model is not capable of in its current form. Another aspect is the model size, where ours is significantly larger than the *Segmentor* and requires more computational resources. (Fig. 5).

### Studying sub-second dynamics and site-specific properties

**Fig. 6:**
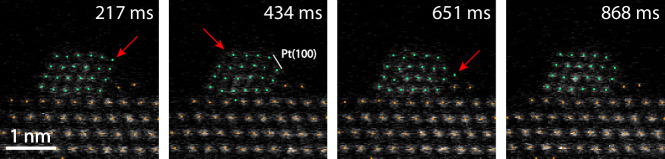
Sub-second dynamics. Selected frames from a time series of a small Pt particle supported on CeO_2_(111). In the first frame our model locates a Pt atom in the top right corner of the particle, situated on what we estimate to be a 2 × 3 Pt(100) facet. The second frame shows reconfigurations taking place in the top left corner of the particle. In the third frame a Pt atom has moved onto the neighboring CeO_2_ step but disappears before the next frame.

Combining both the localization and segmentation allows us to study column movements, or even single atom movements if the object is sufficiently thin, with sub-second temporal resolution and sub-pixel precision. We applied our model to a time series of a small Pt nanoparticle estimated to be 118 atoms large^1^, supported on CeO_2_(111) by a small step edge. A selected sequence is presented in Fig. 6. We can see how the particle is constantly rearranging under electron irradiation, with Pt atoms moving for example, on and off of exposed Pt(100) facets or onto the nearby CeO_2_ step.

**Fig. 7:**
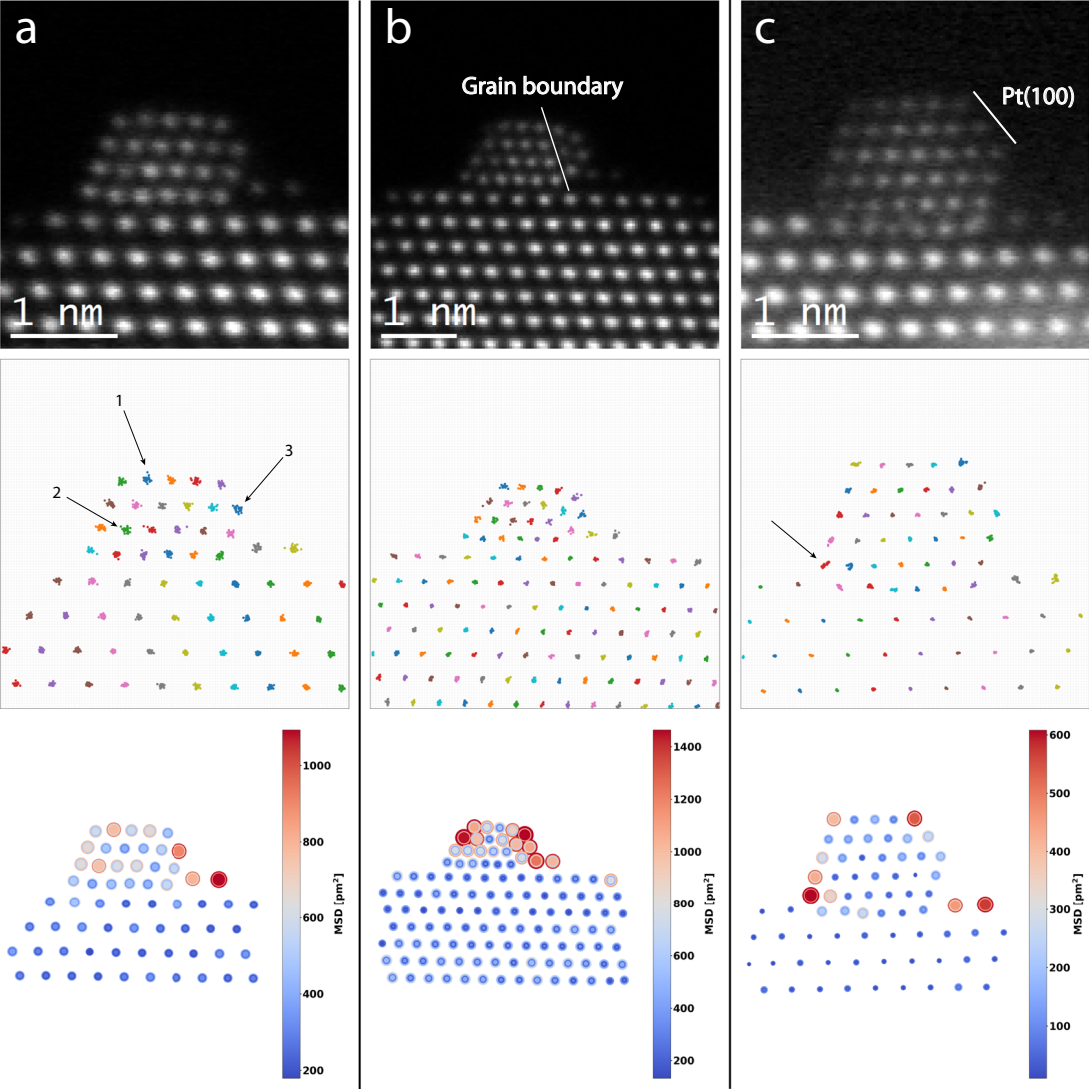
Site-specific dynamics of nanoparticle columns. Three particle structures were studied by tracking the movement of their atomic columns throughout time series. For each particle, we present a summed image of the entire time series, a color-coded plot displaying the linked column positions, and a diagram of the mean square displacement (MSD) of each atomic column over the time series. The solid circle represents the mean square displacement and the inner and outer circles represent MSD ± ΔMSD. **a** A stable particle structure imaged ex situ, the time series comprises 50 frames out of which 27 frames where the localization model predicts the same structure were used to link the column positions and calculate the MSD. Three sites of elevated dynamics are pointed out. **b** A relatively unstable particle structure containing a grain boundary. The structure was imaged ex situ over 35 frames out of which 13 were used for the subsequent analysis. High MSD values can be observed in the top left corner and on the outer side of the grain boundary which is in agreement with dynamics that can be observed in the supporting movies. **c** A particle imaged in situ (see methods). We used 36 frames, where each frame was formed as the sum of itself and the next 19 consecutive frames from the original time series to track the column positions and calculate the column MSD. The highlighted interface Pt column appears to have been oscillating along an axis directed from the particle center towards the closest CeO_2_ column, hinting at Pt–CeO_2_ interaction in the 10% H_2_/90% N_2_ atmosphere.

We can also utilize our model to visualize the movements of individual columns over time to reveal information about specific sites. Two particle structures observed ex situ and one observed in situ were selected for analysis. From the original time series, we select the frames where the output of our model contains all expected column positions. It should be noted that the frames from the in situ series are averaged over 20 consecutive frames while the ex situ frames are as-recorded. Then, these frames are linked in time, connecting each column position with itself throughout the time series. Positions are linked by assigning the closest position within 5 pixels of the position in the previous frame as the new position. We then calculate the mean square displacement of each linked position and the result is visualized in Fig. 7.

As our localization precision is dependent on signal strength (Fig. 3b), there could be a thickness dependency in the column mean square displacement (MSD). If sample thickness was the most significant factor in observed differences in MSD, the pattern should align with the column intensities due to the mass-thickness contrast of the HAADF signal. Furthermore, since single atom movements in thin columns would impact the resulting position of the projected atomic column more than the same movement in a thick column, there is likely a bias towards higher dynamics in thinner columns. The magnitude of these effects is hard to estimate. However, given that the MSD values are reliable, assuming a localization error of 0.5 pixels, which corresponds to the performance of the localization model on very noisy simulated data (Fig. 3), we can point out sites where thickness effects are unlikely to be the explanation for an elevated MSD value.

If we estimate the particle in Fig. 7a to have a truncated octahedral shape (Supplementary Fig. 8), the highlighted columns 1 and 2 should be 4 and 6 atoms thick, respectively^1^. Yet, they both exhibit a similar MSD which is elevated with respect to their thinner and thicker neighboring columns. Thus we estimate that the thickness effect is small in this case. An interesting aspect of column 1 is that in several frames of the complete time series, it is split into two separate column with one on top of the other as seen in Supplementary Fig. 9. This further supports the claim that there are actual reconfigurations taking place at this site and that it is reflected in the column mean square displacement. Another example is the highlighted column “3” which should have a similar thickness to its neighboring corner column, yet it displays a much larger MSD. This could be related to the commonly observed movement of Pt atoms onto the adjacent CeO_2_, as displayed in Fig. 6.

In Fig. 7b we present a particle structure with a small grain boundary. In this case, the columns on the right side of the boundary exhibit high MSD values along with the columns in the top left corner of the particle. This agrees with the restructuring that can be observed in the experimental time series (see Supplementary Videos).

Finally, the particle observed in situ in 10% H_2_/90% N_2_ at 1000 mbar and 300 °C is presented in Fig. 7c. This particle is significantly larger than the previous particles and there are few distinctive features in the MSD plot. One interesting observation is that the linked positions of the column in the bottom left corner form a line, indicating that the column has oscillated along this axis extending from the center of the Pt particle to the nearest CeO_2_ column. This pattern suggests an interaction with the support which highlights that this technique could be of use in heterogeneous catalysis where interface sites and fluxional behavior under reaction conditions are believed to play a significant role^36–38^.

## Methods

### Machine learning and data analysis

The machine learning models were implemented with PyTorch^39^ and data analysis was done in Python using homemade functions and various libraries^40–44^. Training and evaluation of the models was done on a workstation equipped with an NVIDIA RTX 4090 GPU, an Intel Core i9-13900K CPU, and 64 GB RAM. Model training took about 40 min for the localization network and just short of 3 h for the segmentation network.

### Experimental data

The experimental ex situ time series are extracts from the comprehensive data presented in our previous work^1^. The raw data were recorded in 512 × 512 frames with a probe-corrected Titan Themis operated at 300 kV. Settings used were: pixel size of 25.36 pm, convergence angle of 18.5 mrad, beam current of 20 pA, and a pixel dwell time of 500 ns. For the presented time series, a 256 × 256 region around the Pt nanoparticle was cut out and rigidly aligned using SmartAlign^45^. The time series were then cropped again around the Pt nanoparticle to a frame size of 128 × 128.

In situ gas-cell time series were recorded with the DENSsolutions Climate holder. A gas mixture of 10% H_2_/90% N_2_ was used. The pressure in the nanoreactor was set to 1000 mbar, the flow was 0.15 ml/min, and the temperature was set to 300 °C. The imaging parameters were the following: a pixel size of 25.38 pm, a convergence angle of 18.5 mrad, a beam current of 64 pA, and a pixel dwell time of 500 ns. Post-processing followed the same procedure as described above.

### Supplementary information

Supplementary Information

Supplementary Video 1

Supplementary Video 2

Supplementary Video 3

## Data Availability

Model weights and data used to train and evaluate the models can be found on Zenodo^46^.

## Code availability

The PyTorch implementation of the models is available on GitHub: https://github.com/benke97/ParticleLocSeg.
