# Supplementary material for: Localization and segmentation of atomic columns in supported nanoparticles for fast scanning transmission electron microscopy
Source: NPJ Comput Mater. 2024 Aug 3;10(1):168. doi: 10.1038/s41524-024-01360-0 (PMC11297796; doi:10.1038/s41524-024-01360-0)
Supplement: Supplementary file 1 — Supplementary Information [file 41524_2024_1360_MOESM1_ESM.pdf]

# Supplementary Information

## Localization and Segmentation of Atomic Columns in Supported Nanoparticles for Fast Scanning Transmission Electron Microscopy

Henrik Eliasson[1] and Rolf Erni[1, 2*]

*[1]Electron Microscopy Center, Empa – Swiss Federal Laboratories for Materials Science and Technology, Überlandstrasse 129, 8600 Dübendorf, Switzerland.*

*[2]Department of Materials, ETH Zürich, CH-8093 Zürich, Switzerland.*

## Mean square displacement error

The error of the mean square displacement was calculated by propagating the mean positional error of the localization network. Each displacement $d_{\mathrm{i}}$ between points $p_{\mathrm{new}}$ and $p_{\mathrm{current}}$ is calculated as

$$d_{\mathrm{i}} = p_{\mathrm{new}} - p_{\mathrm{current}}$$

with corresponding displacement error

$$\Delta d_{\mathrm{i}} = \sqrt{(\Delta p_{\mathrm{new}})^2 + (\Delta p_{\mathrm{current}})^2} = \sqrt{2}\epsilon$$

where $\epsilon$ is the mean positional error of points predicted by the localization network. The expression for the error of the squared displacement, $d_{\mathrm{i}}^2$, is then obtained through error propagation as

$$\Delta d_{\mathrm{i}}^2 = 2\sqrt{2}\epsilon|d_{\mathrm{i}}|.$$

The mean squared displacement is expressed as

$$\mathrm{MSD} = \frac{1}{N}\sqrt{\sum_{i=1}^{N} d_i^2}$$

where N is the number of points in the sequence, with corresponding MSD error derived as

$$\Delta\mathrm{MSD} = \frac{1}{N}\sqrt{\sum_{i=1}^{N} (\Delta d_i^2)^2} = \frac{1}{N}\sqrt{\sum_{i=1}^{N} \left(2\sqrt{2}\epsilon|d_i|\right)^2}\ .$$

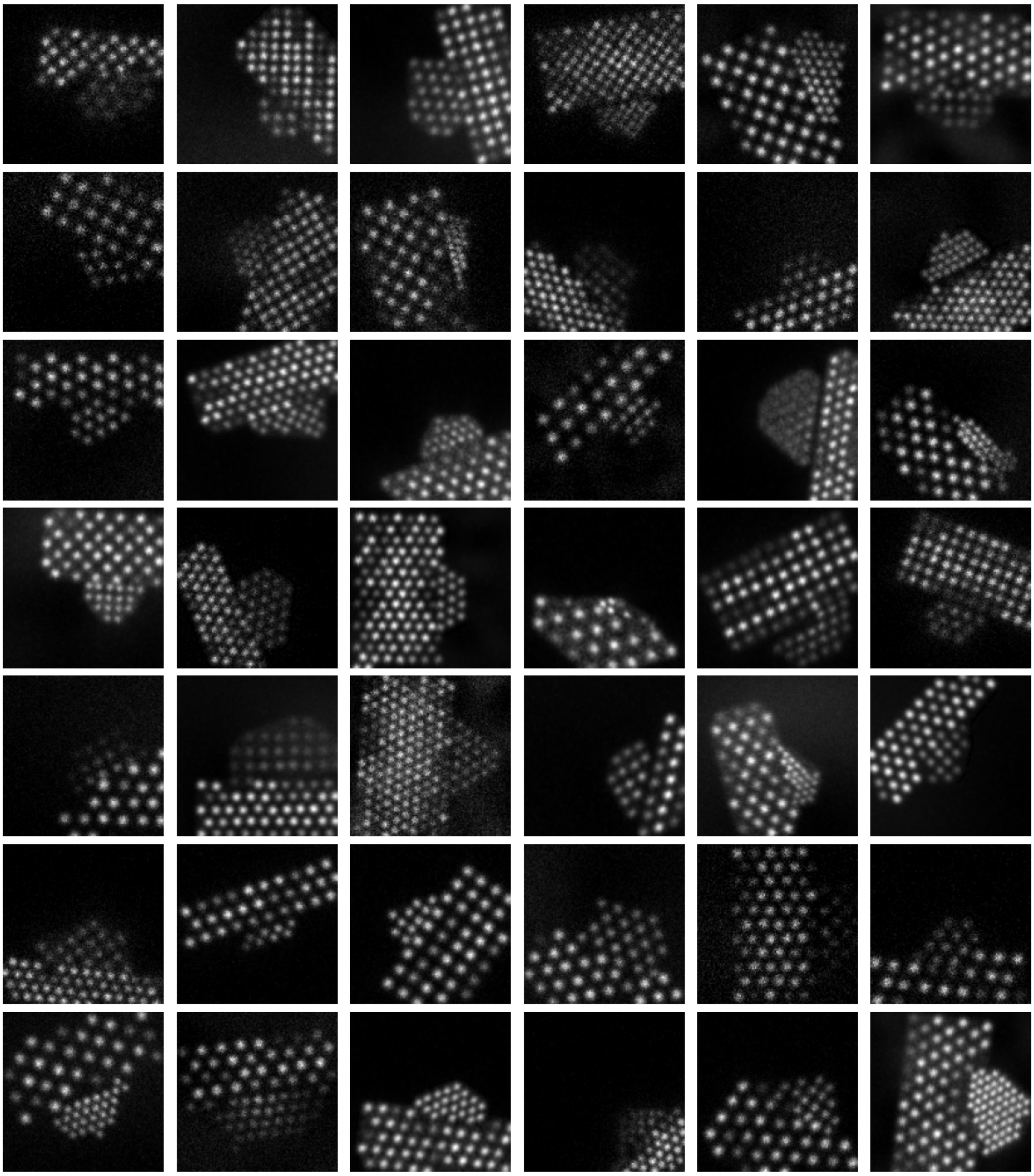

**Supplementary Figure 1** An extract of 42 randomly selected images from training dataset.

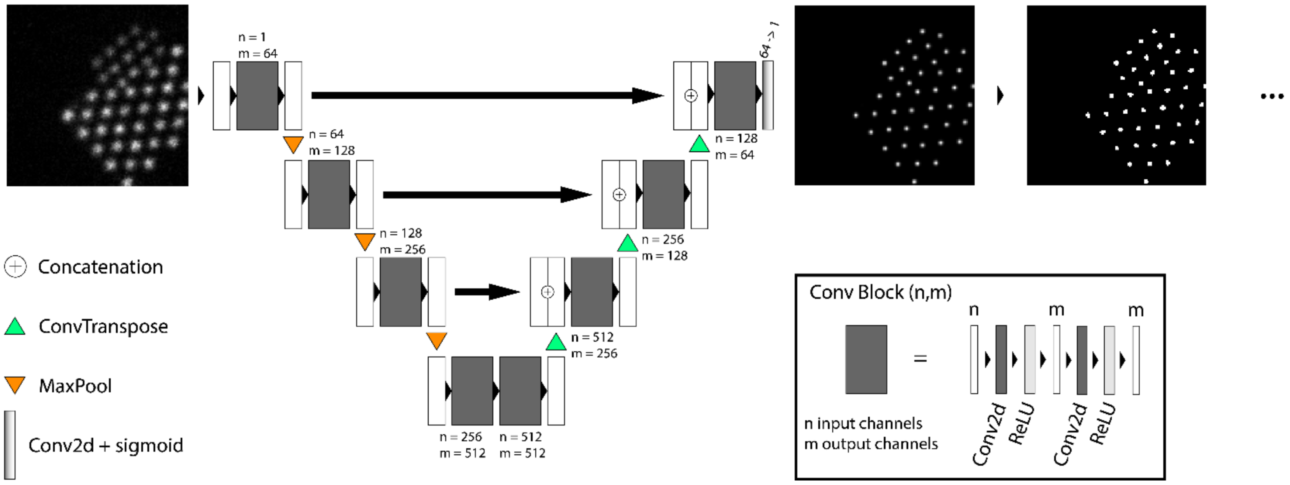


**Supplementary Figure 2** Schematic of the localization network and related workflow.

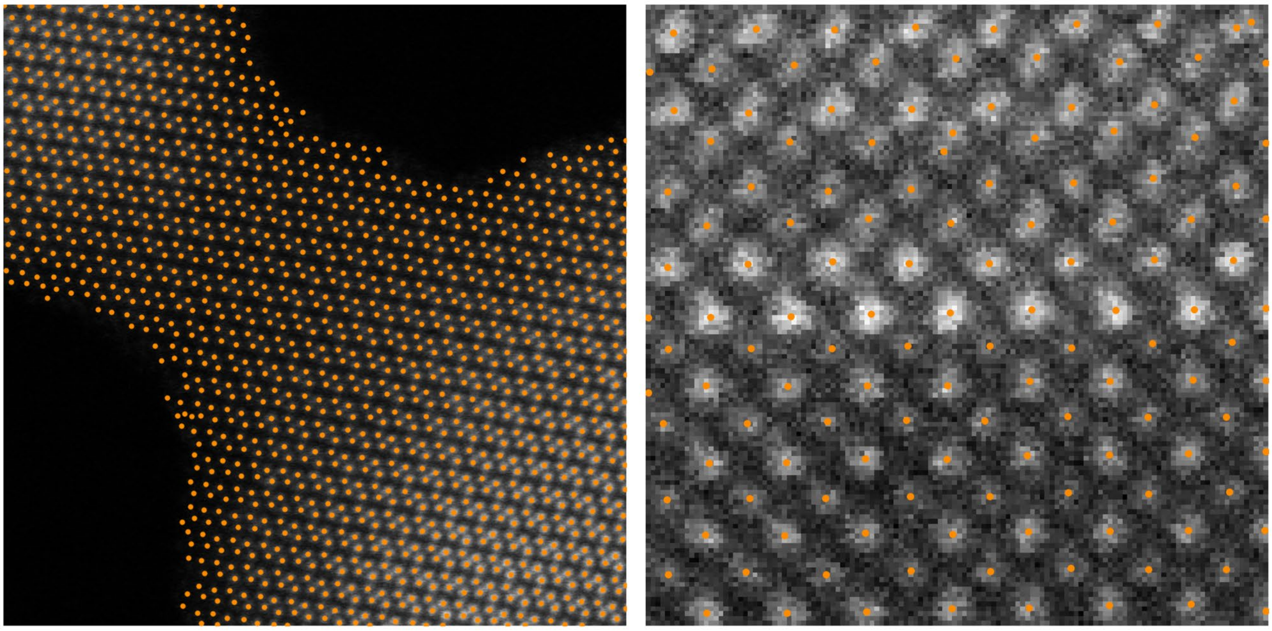

**Supplementary Figure 3** The model applied to (left) a 1024x1024 image of monoclinic $ZrO_2$, a much larger image size than that of the training data, as well as a lattice geometry that the model was not trained on. (right). An HR-STEM image from the TEAM microscope of a perovskite interface.

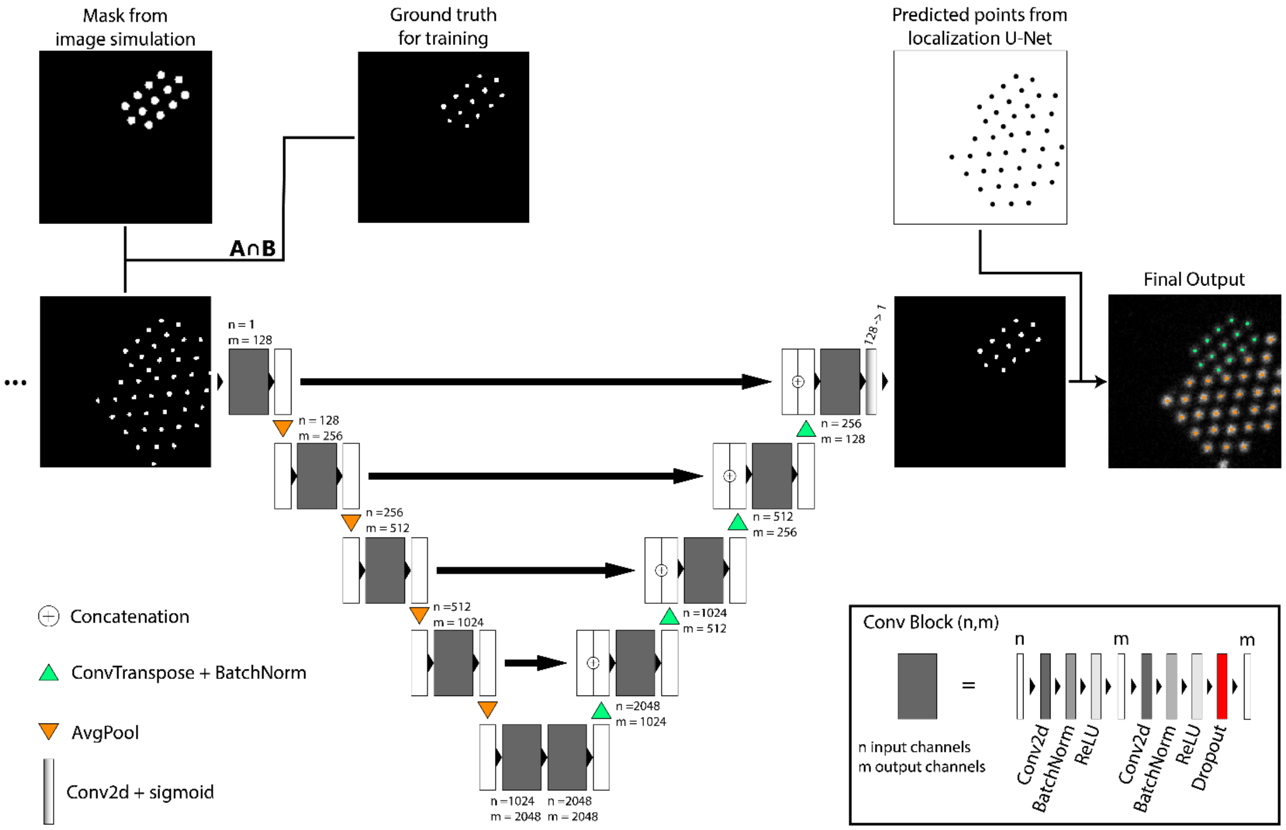


**Supplementary Figure 4.** Schematic of the segmentation network and related workflow.

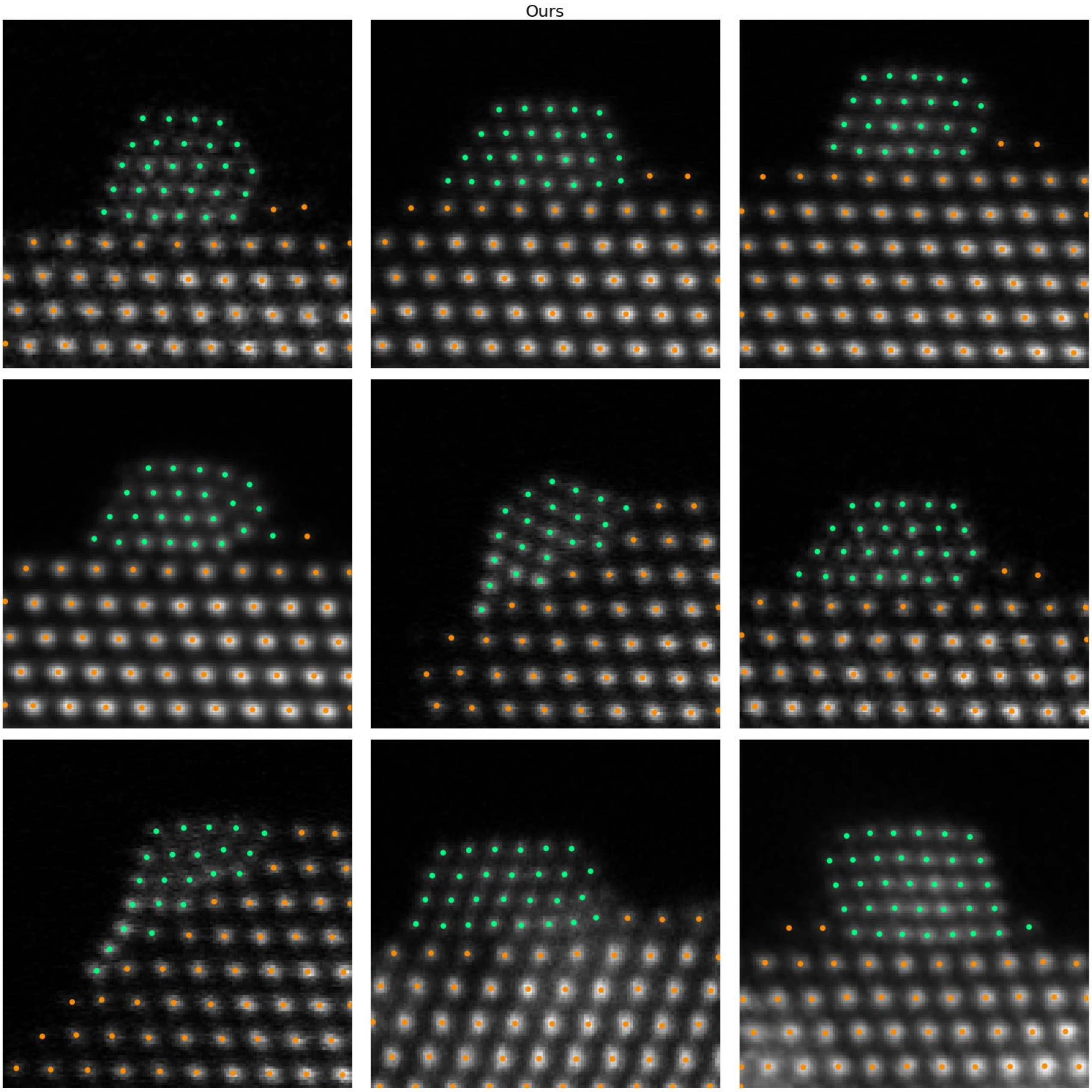


**Supplementary Figure 5** The combined localization and segmentation model applied to a test set of experimental images.

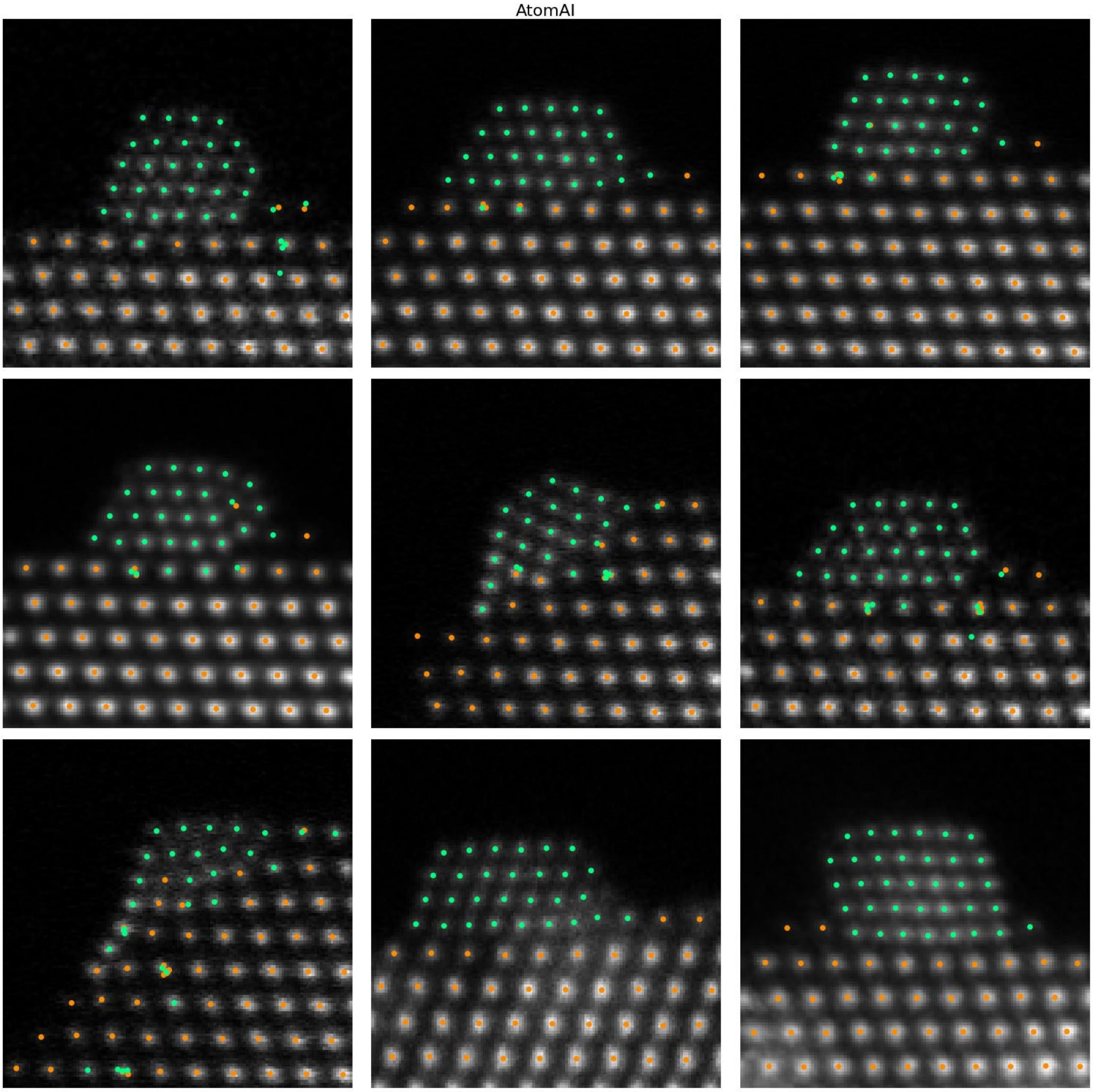


**Supplementary Figure 6** AtomAI's *Segmentor* model applied to a test set of experimental images.

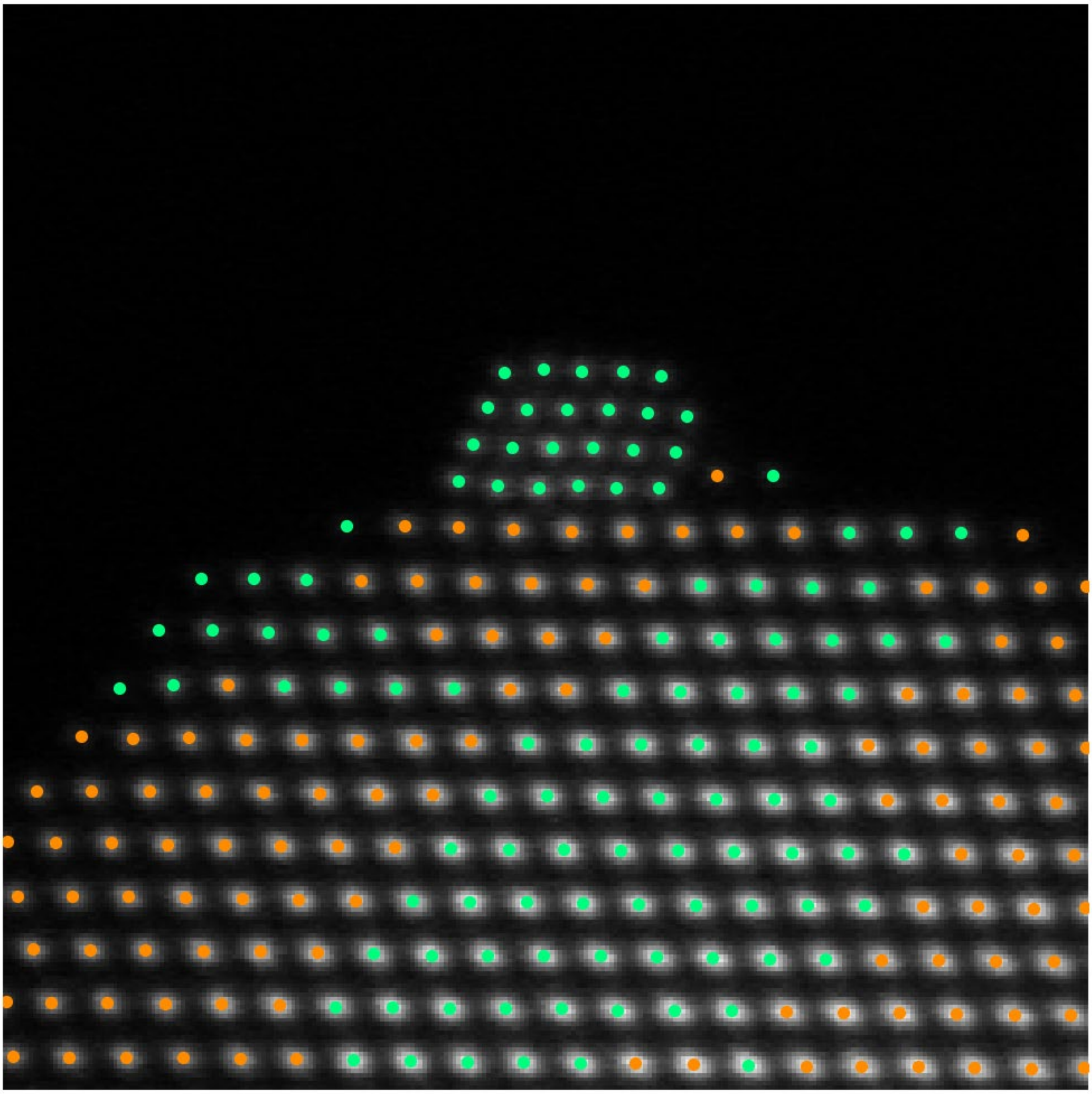

**Supplementary Figure 7** Our model applied to a 256x256 particle-support interface image. Although the localization model finds all columns, the segmentation model does not generalize well to larger input sizes than 128x128.

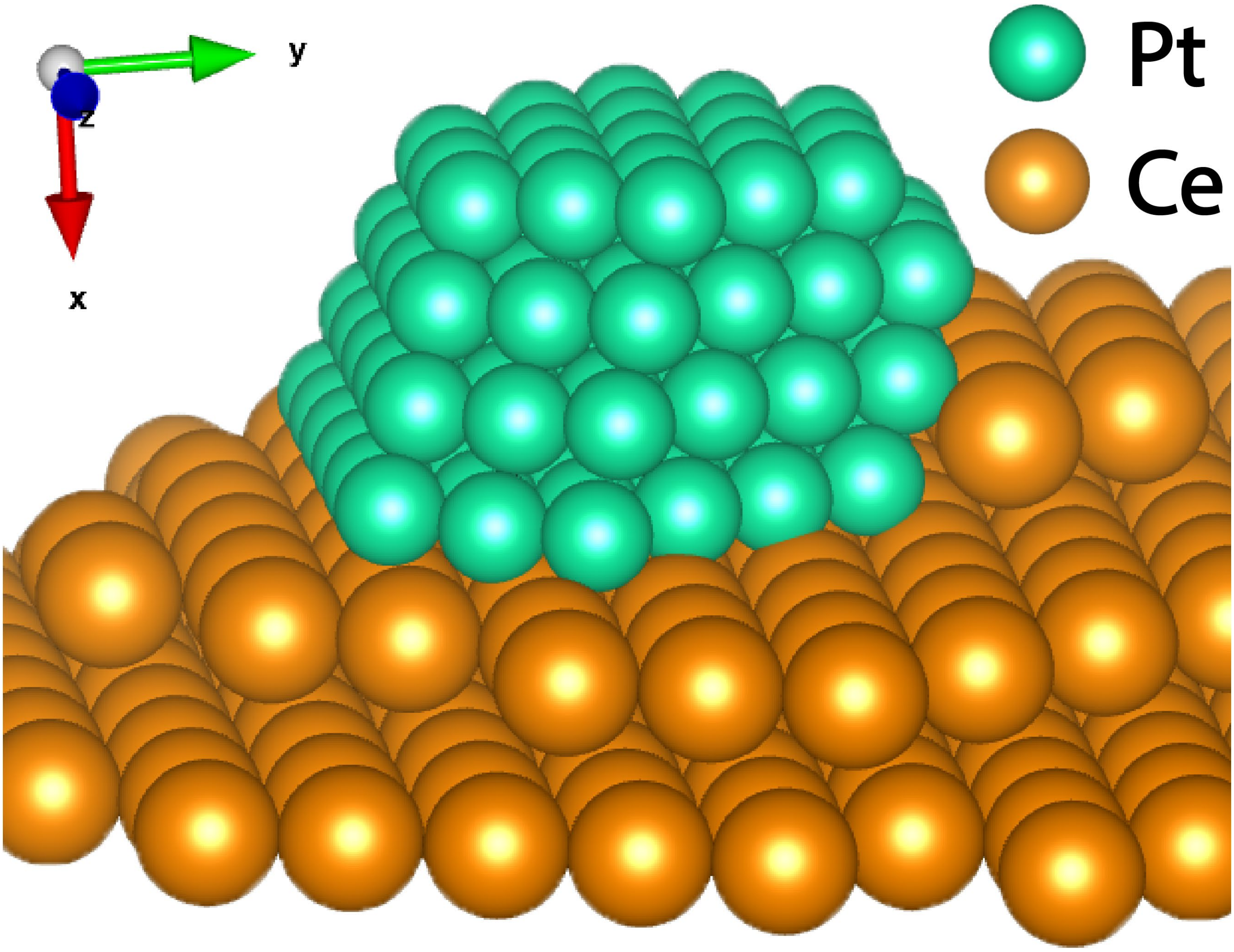


**Supplementary Figure 8** The Predicted particle structure of the particle in Fig. 7a**.** The estimated structure is inspired by a truncated octahedron and is built up of 118 Pt atoms.

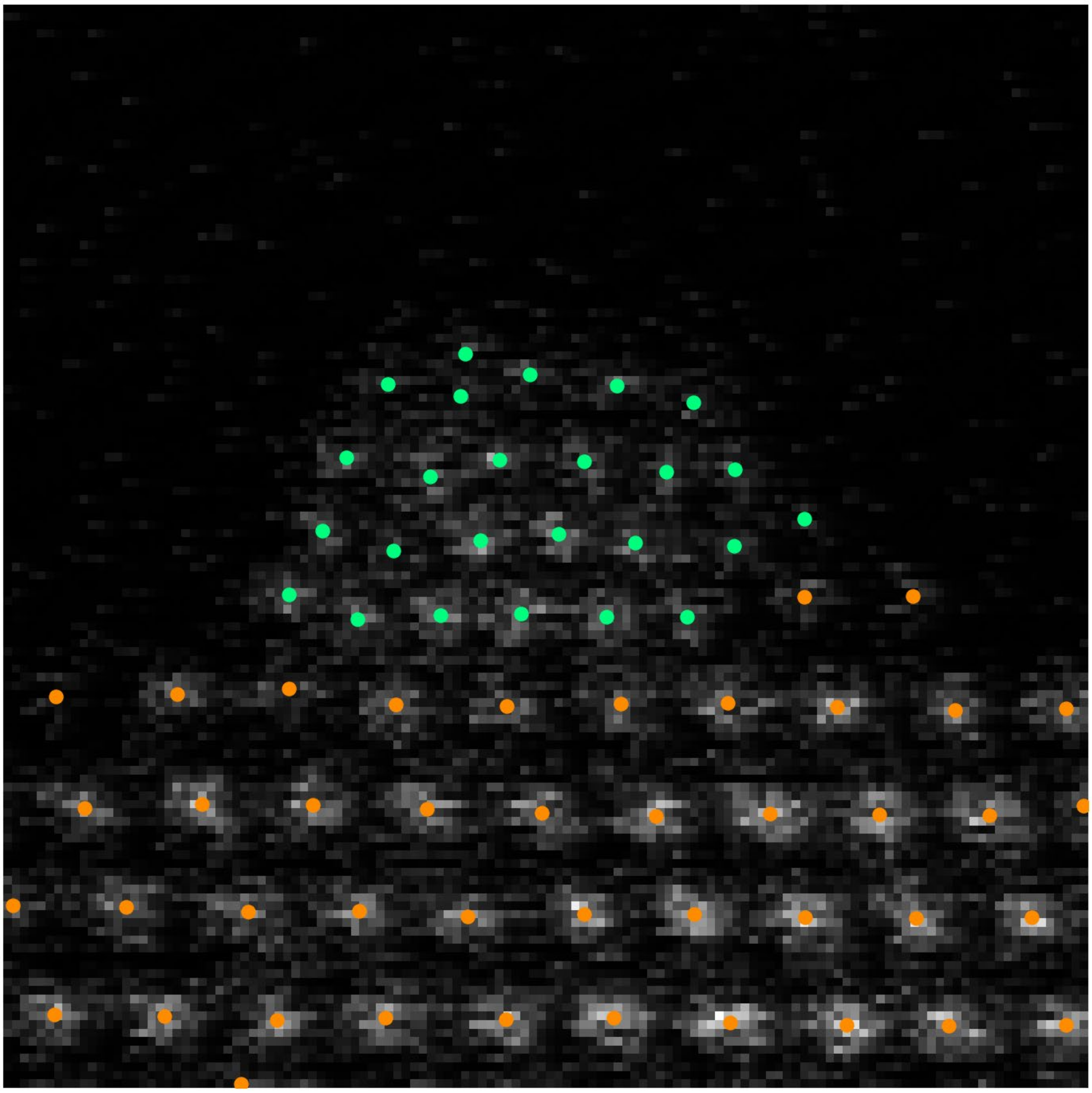

**Supplementary Figure 9** Column splitting in one of the sites exhibiting a high mean square displacement. The column to the right of the top left corner has split into two, this behavior can be observed more than once in the time-series. This site in Fig. 7 of the main text exhibits a high MSD which is likely correlated to the splitting.

**Supplementary Video 1** The raw time-series of the structure in Figure. 7a of the main text. The time-series was recorded at 5 frames per second and is played back at 20 frames per second.

**Supplementary Video 2** The raw time-series of the structure in Figure. 7b of the main text. The time-series was recorded at 5 frames per second and is played back at 20 frames per second.

**Supplementary Video 3** The in-situ time-series of the structure in Figure. 7c of the main text. Each frame is the sum of itself and the next 19 frames. The time-series was recorded at 5 frames per second and is played back at 20 frames per second.
