# Supplementary figures and images for: Localization and segmentation of atomic columns in supported nanoparticles for fast scanning transmission electron microscopy
Source: NPJ Comput Mater. 2024 Aug 3;10(1):168. doi: 10.1038/s41524-024-01360-0 (PMC11297796; doi:10.1038/s41524-024-01360-0)

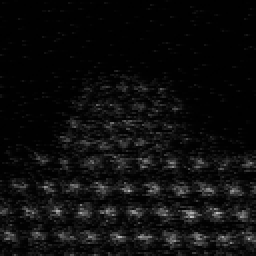

Supplement: Supplementary file 2 — Supplementary Video 1 [file 41524_2024_1360_MOESM2_ESM.gif]

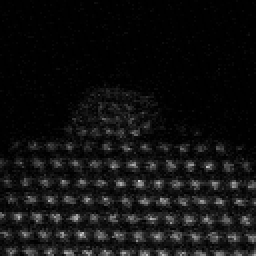

Supplement: Supplementary file 3 — Supplementary Video 2 [file 41524_2024_1360_MOESM3_ESM.gif]

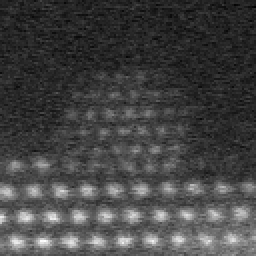

Supplement: Supplementary file 4 — Supplementary Video 3 [file 41524_2024_1360_MOESM4_ESM.gif]
